# Book Review: Braastad et al. *The Cat: Behaviour and Welfare*; CABI: Wallingford, UK, 2022; ISBN: 978-1789242317

**DOI:** 10.3390/ani14162409

**Published:** 2024-08-20

**Authors:** Eugenia Natoli

**Affiliations:** Canile Sovrazonale, ASL Roma 3 (Local Health Unit Rome 3), 00148 Rome, Italy; enatoli@tiscali.it

It was challenging to read the entire book *The Cat: Behaviour and Welfare* by Bjarne O. Braastad, Anne McBride and Ruth C. Newberry because it is a full-bodied and massive book, rich in topics, information, advice, and hints for expanding knowledge on the subject through further reading. The project is ambitious: to take stock of the knowledge of everything concerning the life of the domestic cat, in the environment this species shares with human beings, since the beginning of its history with them, i.e., since domestication.

The book consists of 14 chapters dealing with many topics, ranging from the origin of the domestic cat through to the development of kittens and their relationship with their mother and siblings, cats’ personality, cats’ language—communication, social behaviour, predatory behaviour, and emotions, cats’ welfare, cats’ learning and training, and problem behaviours in cats, with the last part dedicated to the inter-specific interactions between people and cats.

It is sufficient to peruse the chapter titles to realise that the book is not about the behaviour of the domestic cat as a species but rather about its behaviour as a companion animal, i.e., in relation to its relationship with humans. This approach was probably influenced by the authors’ scientific background, which is more oriented towards applied ethology than to behavioural ecology. Not that this is a criticism of the approach; it is probably a more useful approach for a cat owner. Owners need to have advice on how to manage cats living in their homes to ensure that they get along with each other rather than to know, in theory, how a social group of cats living outside humans’ sphere of influence is organised. Nevertheless, having knowledge about the social organisation of a group of domestic cats managing inter-individual relationships without human interference can help people to understand what happens in their own homes.

Despite the book providing a considerable amount of information, it often lacks an explanation of the function of behaviours. By function, we mean “what a behaviour is for” or why natural selection has favoured it in the original environment of evolution (the open bushland around the first human settlements?). The answer is that it provided advantages for the survival and reproduction of the individual displaying it. It was so advantageous that domestic cats, living in an environment (such as an apartment) completely different from that of their ancestors (the open bushland), still exhibit it. All behaviours have, or have had, a function. If the behaviour is no longer effective in achieving a certain goal and we still observe it in our cats, such as scratching with their front paws on the floor (which has replaced grass and soil) to cover their faeces, it means that it does not produce negative effects and therefore remains in the behavioural repertoire of the species. What is missing in the book is a more “biological” perspective of the cat as an animal.

In some chapters, the bibliographic references are not as good because they are sometimes outdated and should have been replaced by more recent publications; furthermore, some references are probably from scientific journals that are too niche for mainstream readers. On the other hand, the book's target readership is listed as animal behaviourists (what does this mean? Ethologists?), veterinary nurses, veterinary professionals, and cat owners. It is impossible to provide reading material that is utilisable for both professional categories and cat owners, who have completely different levels of knowledge.

However, if I had to choose which category to recommend this book to, I would probably choose professionals such as behavioural biologists and veterinarians, because they have more tools to understand it and critically choose which parts are most valuable and which advice to follow.

More specifically, these points of criticism mainly concern certain chapters. The first two chapters, which deal respectively with the origin and ontogenetic development of the domestic cat, are rather outdated and sometimes inaccurate and obvious. The terminology utilised (e.g., stray) is old and the bibliography limited. The authors use popular but, perhaps, sometimes too technical language.

Even more problematic are Chapters 4 and 5, which give an overview of the language and communication (visual, olfactory, and acoustic) and social behaviour of the domestic cat. Unfortunately, there are only a few further recommended readings, which are really dated. It is true that Leyhausen was the scholar who paved the way, but from 1979 (the date of publication of his book in English, which was originally published in 1973 in German) to the present day, knowledge has expanded and enriched. This choice by the authors gives rise to many errors that run throughout both chapters. Many things they state have been refuted due to new studies and are therefore, quite simply, wrong. The peak is reached when the authors state, without bibliographic support, that in the African wildcat, *Felis lybica*, ancestor of the domestic cat, the male and female sometimes live together in monogamy. If one thing is certain, it is that the wild cat, European or African, is not monogamous. It is definitely polygynous, especially in origin.

The same problem is found in Chapter 9 (Animal welfare—how to ensure the cat’s welfare), with some mistakes that worsen the situation. For example, due to a general confusion of definitions, authors state that “…kittens born to homeless mothers and raised with little or no contact with people might be referred to as wildcats, but this is not possible because true wildcats belong to a different species from the domestic cat”. From a zoological point of view, this is a big mistake given that there is exhaustive literature yielding evidence that wildcats and domestic cats are subspecies, not different species. Moreover, without distinguishing between one country and another and without providing the necessary bibliographical references, the authors report that, if they survive, female ownerless cats can have three to four litters a year. Considering that in Italy, with its mild climate, females do not always manage to have two litters a year, it is fanciful that in Northern Europe they manage to have three or four.

Chapter 10 is on ‘Learning and training’ in domestic cats. It gives a good overview of these issues, although it lacks the vision of behavioural ecology, i.e., a framing of the described behaviours in the broader context of the function of the behaviours, i.e., why they evolved.

The eleventh chapter on behavioural problems in cats begins correctly by stating that often “problematic” behaviours are problematic for us and not for the cat. Subsequently, however, the authors contradict themselves, for example, when they address the problem of aggressive behaviour since they do not explain that aggressive behaviour is part of the behavioural repertoire of the species and that it serves the individual to acquire the resources necessary for its survival. They do not say that only if excessive aggressive behaviour is pathological, we should give advice on how to repress it. They assume all aggressive behaviour is pathological. An ethologist, or a veterinarian, would certainly have more tools to understand the advice given in the book, i.e., when aggressive behaviour, qualitatively and quantitatively, can be attributed to a behavioural problem, when it is appropriate to treat it with drugs, and when it is not. Finally, I think that a scientific/popular book should avoid advertising private industries that produce synthetic pheromones, naming products for which the chemical composition has not even been scientifically validated.

In contrast, as far as some topics are concerned, this book is a good manual! The best chapters are the third, the sixth, the seventh and the eighth because they are based on scientific evidence reported in the text.

The third chapter deals with inter-individual differences between cats, i.e., different personalities. But it does not limit itself to this; it also reports how the different personalities of the owners influence those of their cats, supporting the conclusions with bibliographical evidence. Furthermore, it emphasises the influence of two other extremely important variables, the cat's age and sex, on personality. Another issue that the third chapter affords is the effect of age on a cat's behaviour. Also, cats can suffer from senile dementia, and the authors emphasise that cats displaying certain behaviours that are disreputable to humans, such as urinating outside the litter box, are not out of ‘spite’ as is often commonly believed but rather deviant behaviour due to advanced age. Finally, the chapter does not overlook the effect of genetics and the cat's developmental environment on its personality.

Chapter 6 is the one I preferred most. The description of the domestic cat as a predator, the contradictions caused in the Anthropocene by its role, which is why it was domesticated, and a broad overview of the pros and cons of its predatory behaviour, and the means to contain it, increase the reader’s knowledge on the subject.

Stories of lost cats who return home after days or even months have always fascinated humans. Chapter 7 deals with the subject and describes in an excellent way the hypotheses formulated by scholars to explain the phenomenon.

Chapter 8 is equally appreciated because it deals with the sensitive subject of emotions in cats, both positive and negative. Its value lies in the fact that it makes it clear that cats feel emotions and that they manifest them whether they are stressed or relaxed. It would seem to be a matter of course, but not all people, paradoxically even among insiders, believe that animals can feel emotions and that they can have important consequences for the behaviour of cats, even in the long term. There is probably too much optimism on the part of the authors in describing, and considering, stereotypies as degenerative display activities, which they are not. That would be nice, but unfortunately, this is not the case; otherwise, stereotypies would be more easily resolved. The cerebral pathway of mediators leading to stress-related behaviour is different for displaying activities and stereotypies.

The last three chapters fall into the general idea of providing a handbook for stakeholders, useful for managing their cat(s). Chapter 14 is rather a summary of the book than a set of conclusions.

The chapters are accompanied by agreeable images and the graphics of this book are very attractive.

